# Supplementary material for: Assessment of Endometriosis Knowledge and Its Determinants Among Nurses in Al-Jouf Region, Saudi Arabia
Source: Healthcare (Basel). 2025 Jun 11;13(12):1386. doi: 10.3390/healthcare13121386 (PMC12193512; doi:10.3390/healthcare13121386)
Supplement: Supplementary file 1 [file healthcare-13-01386-s001.zip › healthcare-3665920-supplementary.pdf]

Assessment of Endometriosis Knowledge and Its Determinants Among Nurses in Al-Jouf Region, Saudi Arabia

Part One (Demographic and Professional Data)

1. **Age:**
    1. 23-28 years
    2. 29-34 years
    3. 35-40 years
    4. 41-46 years
    5. 47-52 years

2. **Educational level:**
    1. Nursing diploma
    2. Bachelor's degree in Nursing
    3. Master's degree in Nursing or more

3. **How many years of nursing/gynecology experience?**
    1. Less than one year
    2. 1- 4 years
    3. 5- 10 years
    4. 11- 15 years
    5. 16-20 years
    6. More than 20 years

4. **Have you previously received training courses in caring for patients with endometriosis?**
    1. Yes
    2. No

5. **Have you ever cared for patients with endometriosis?**
    1. Yes
    2. No

The second part: Nurses' Knowledge about Endometriosis

6. **What is endometriosis?**

    1. The descent of the uterus into the vagina
    2. When the rectum protrudes through the back wall of the vagina
    3. When uterine tissue begins to grow in areas outside the uterus
    4. A case of weak pelvic muscles

7. **In what percentage of women does endometriosis occur?**

    1. 0-15%
    2. 16-30%
    3. 31-45%
    4. 46-60%
    5. 61-80%

8. **What are the risk factors for endometriosis? Select all that apply.**

    1. Early onset of menstruation
    2. Late onset of menopause
    3. Having many children
    4. Low body mass index
    5. Family history of endometriosis

9. **When are most cases of endometriosis diagnosed?**

    1. Between the ages of 12-14 years
    2. Between the ages of 15-21 years
    3. Between the ages of 25 - 35 years
    4. Between the ages of 40-50 years

10. **What diagnostic test is considered the gold standard for confirming or rejecting the diagnosis of endometriosis?**

    1. Clinical evaluation.
    2. Vaginal ultrasound
    3. Magnetic resonance imaging
    4. Laparoscopy

11. **Does an endometriosis patient suffer from excruciating pain during her menstrual cycle?**
    1. Yes
    2. No

12. Clinical manifestations of endometriosis include: (Please select all that apply).

1. **Abdominal pain**
2. **Chronic pelvic pain**
3. **Urinary incontinence**
4. **Pain during or after intercourse**
5. **Pain with bowel movements**
6. **Premenstrual spotting or bleeding between menstrual cycles**
7. **painful bladder syndrome**
8. **Heavy bleeding during menstruation**

13. Infertility is one of the most disturbing symptoms of endometriosis.

1. **Yes**
2. **No**

14. Which of the following mental health disorders is most frequently associated with endometriosis?

1. **Bipolar disorder**
2. **Depression**
3. **Paranoid personality disorder**
4. **Obsessive-compulsive disorder**

15. What is the goal of treating endometriosis?

1. **Reducing pain and maximizing the ability to reproduce**
2. **Reducing the feeling of pelvic fullness**
3. **Reducing the occurrence of stress and pain**
4. **Increase the strength of the pelvic floor muscles**

16. Which of the following classes of drug treatments are commonly prescribed for endometriosis? (Select all that apply).

1. **Non-steroidal anti-inflammatory drugs**
2. **Hormonal contraceptive pills**
3. **Thiazide diuretics**

17. When women suspect endometriosis, what information do they base their assumptions on? (multiple answers possible)

1. **Health center/health professionals (doctor, nurse, pharmacist)**
2. **The Internet and social media**
3. **Media - TV/radio/newspapers**
4. **Relatives/friends**
5. **Magazine/book/brochure**

تقييم المعرفة ببطانة الرحم المهاجرة والعوامل المحددة لها بين الممرضات في منطقة الجوف بالمملكة العربية السعودية

الجزء الأول (البيانات الديموجرافية والمهنية)

1. العمر :

1. 23- 28 عاما"
2. 29- 34 عاما"
3. 35-40 عاما"
4. 41-46 عاما"
5. 47-52 عاما"

2. مستوى التعليم:

1. دبلوم تمريض
2. بكالوريوس تمريض
3. ماجيستير تمريض أو اكثر

3. كم عدد سنوات الخبرة في التمريض / أمراض النساء؟

1. أقل من سنة
2. 1- 4 سنوات
3. 5- 10 سنوات
4. 11- 15 سنوات
5. 16- 20 سنة
6. أكثر من 20 سنة

4. هل سبق الحصول على دورات تدريبية في رعاية المرضى الذين يعانون من بطانة الرحم المهاجرة؟

1. نعم
2. لا

5. هل سبق ان قمتي برعاية المرضى الذين يعانون من بطانة الرحم المهاجرة؟

1. نعم
2. لا

الجزء الثاني معلوماتك عن بطانة الرحم المهاجرة

6. ما هي بطانة الرحم المهاجرة ؟
1. نزول الرحم إلى المهبل
2. عندما يبرز المستقيم من خلال الجدار الخلفي للمهبل
3. عندما يبدأ نسيج الرحم بالنمو في مناطق خارج الرحم
4. حالة من ضعف عضلات الحوض

7. في أي نسبة من النساء تحدث بطانة الرحم المهاجرة؟
1. 0-15%
2. 16-30%
3. 31-45%
4. 46-60%
5. 61-80%

8. ما هي عوامل خطر الإصابة ببطانة الرحم المهاجرة ؟ اختر كل ما ينطبق.
1. بداية الحيض في وقت مبكر
2. بداية سن اليأس في وقت متأخر
3. إنجاب الكثير من الأطفال
4. انخفاض مؤشر كتلة الجسم
5. تاريخ عائلي لبطانة الرحم المهاجرة

9. متى يتم تشخيص معظم حالات بطانة الرحم المهاجرة ؟
1. بين سن 12-14 سنة
2. بين سن 15-21 سنة
3. بين سن 25 - 35 سنة
4. بين سن 40-50 سنة

10. ما هو الاختبار التشخيصي الذي يعتبر المعيار الذهبي لتأكيد أو رفض تشخيص بطانة الرحم المهاجرة بشكل مؤكد؟
1. التقييم السريري.
2. الموجات فوق الصوتية المهبلية
3. التصوير بالرنين المغناطيسي
4. تنظير البطن

11. هل مريضة بطانة الرحم المهاجرة تعاني من الام مبرحة أثناء الدورة الشهرية ؟
1. نعم
2. لا

12. تشمل المظاهر السريرية لبطانة الرحم المهاجرة ما يلي: (يرجى تحديد كل ما ينطبق).
1. الام بالبطن
2. آلام مزمنة بالحوض

3. سلس البول
4. ألم أثناء الجماع أو بعده
5. ألم مع حركات الأمعاء- الم اثناء التغوط
6. نزيف ما قبل الحيض أو الم بين الدورات الشهرية
7. متلازمة المثانة المؤلمة
8. غزارة الطمث -نزيف غزير بشكل غير طبيعي في فترة الحيض

13. العقم هو واحد من أكثر أعراض بطانة الرحم المهاجرة المقلقة.

1. نعم
2. لا

14. أي من اضطرابات الصحة العقلية التالية كثيرًا ما يصاحبها مرض بطانة الرحم المهاجرة؟

1. الاضطراب ثنائي القطب
2. الاكتئاب
3. اضطراب الشخصية بجنون العظمة
4. اضطراب الوسواس القهري

15. ما هو الهدف من علاج بطانة الرحم المهاجرة ؟

1. التقليل من الألام وتعظيم القدرة على الإنجاب
2. تقليل الشعور بامتلاء الحوض
3. تقليل حدوث الإجهاد والالم
4. زيادة قوة عضلات قاع الحوض

16. أي من الفئات التالية من العلاجات الدوائية يتم وصفها عادة من أجل بطانة الرحم المهاجرة؟ (اختر كل ما ينطبق).

1. العقاقير غير الستيرويدية المضادة للالتهابات
2. حبوب منع الحمل / الهرمونات
3. مدرات البول

17. عندما تشك النساء في إصابتهن بمرض بطانة الرحم المهاجرة، ما هي المعلومات التي يبنين عليها افتراضاتهن؟ (إجابات متعددة ممكنة)

1. المركز الصحي / المهنيين الصحيين (الطبيب، ممرضة، صيدلي)
2. الإنترنت ووسائل التواصل الاجتماعي
3. وسائل الإعلام - التلفزيون / الراديو / الصحف
4. عن طريق الاتصال بالأقارب/الأصدقاء
5. من مجلة / كتاب / كتيب
